# Contained Rupture of a Saphenous Vein Graft Pseudoaneurysm Following Coronary Artery Bypass Grafting

**DOI:** 10.1155/cric/3203648

**Published:** 2026-07-30

**Authors:** Harry Ramcharran, Mahbub Jamil, Joseph Petro, Zhandong Zhou

**Affiliations:** ^1^ Department of Cardiothoracic Surgery, SUNY Upstate Medical University, Syracuse, New York, USA, upstate.edu; ^2^ Department of Cardiothoracic Surgery, UHS Wilson Medical Center, Johnson City, New York, USA; ^3^ Department of Cardiothoracic Surgery, St. Joseph’s Hospital, Syracuse, New York, USA, sjhc.london.on.ca

## Abstract

Saphenous vein graft (SVG) pseudoaneurysms are a rare cause of late graft failure with potentially lethal consequences if left untreated. We present the case of a patient who underwent multivessel coronary artery bypass grafting more than 10 years earlier and presented with chest pain. She was found to have a large 10 cm pseudoaneurysm involving the SVG‐to‐PDA graft. Workup demonstrated compression of the right ventricle by the pseudoaneurysm, a reduced ejection fraction of 27%, and filling of the pseudoaneurysm from the right coronary system without distal graft filling. Given the patient’s symptoms, imaging findings, and pseudoaneurysm size, urgent resection and revascularization were performed. Postoperatively, ventricular function improved, and despite a complicated hospital course, the patient was discharged to rehabilitation with intact motor function and subsequent resolution of cardiopulmonary symptoms.

## 1. Introduction

Saphenous vein grafts (SVGs) are commonly used conduits for coronary artery bypass grafting (CABG) in patients with hemodynamically significant coronary atherosclerotic disease. Late failure of these grafts is most commonly due to atherosclerotic disease or stenosis at the anastomotic site; aneurysmal dilation and pseudoaneurysm formation are rarer causes but can have dangerous consequences if left untreated. SVG pseudoaneurysms may form at the proximal or distal anastomotic site and can lead to devastating sequelae, including rupture, compression of adjacent structures, fistula formation, and myocardial infarction. We present the rare case of a patient who developed a large 10 cm SVG pseudoaneurysm 10 years after CABG, as well as the operative challenges associated with sternal reentry and excision of the pseudoaneurysm.

## 2. Case Presentation

**Figure 1:**
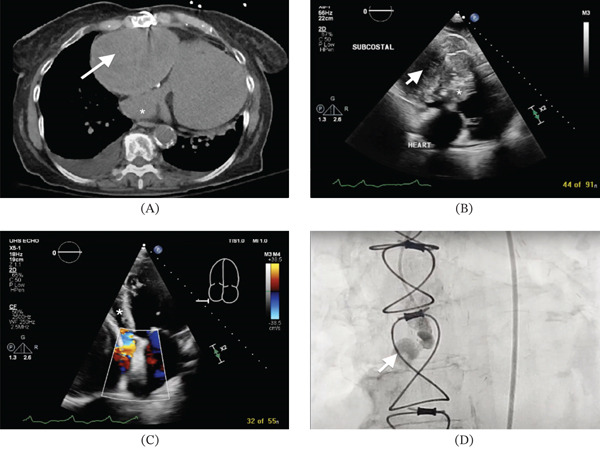
(A) Computed tomography image of the thorax showing a large 10 cm pseudoaneurysm (arrow) compressing the right ventricle (asterisk) and displacing the heart to the left. (B) Echocardiogram with a subcostal view showing a large pseudoaneurysm (arrow) causing compression of the right ventricle (asterisk). (C) Echocardiogram with a four‐chamber view showing right ventricular compression (asterisk) with minimal flow. (D) Coronary angiogram obtained from right coronary injection showing filling of the large pseudoaneurysm (arrow) of the SVG‐PDA conduit with no distal runoff.

A 62‐year‐old female with a past medical history of diabetes mellitus type 2, hypertension, chronic kidney disease stage 4, and coronary artery disease who underwent multivessel CABG 10 years earlier presented to the emergency department with acute‐onset chest pain. Her prior CABG consisted of a left internal mammary artery (LIMA) graft to the left anterior descending artery (LAD) and saphenous vein grafts (SVGs) to the obtuse marginal artery (OM) and posterior descending artery (PDA). Electrocardiography showed no acute ischemic changes, and serial troponin I levels were elevated at 15, 17.1, and 21.2 ng/mL. Serum proBNP was 93,200 pg/mL on admission. Computed tomography of the thorax demonstrated an SVG pseudoaneurysm measuring 10.9 × 8 cm in maximal diameter at the PDA graft body and extending to the distal anastomosis, with a solid component (Figure 1A). Transthoracic echocardiography showed significant compression of the right ventricle with a reduced ejection fraction of 27%, without wall motion abnormalities or hemodynamic instability (Figure 1B). Coronary angiography showed severe native coronary artery disease (Figures 1C and 1D). The LIMA‐to‐LAD graft was widely patent. Evaluation of the right coronary system demonstrated contrast filling of a 10 cm SVG pseudoaneurysm of the SVG‐PDA graft without opacification of the distal PDA (Figure 1D). Given the patient’s acute symptoms, elevated troponin level, and imaging findings, there was concern for compression of the SVG graft by the large pseudoaneurysm, resulting in myocardial ischemia along the PDA distribution. Therefore, given the pseudoaneurysm size, location, and concern for myocardial ischemia, urgent open resection and revascularization were planned. Endovascular exclusion and embolization were considered unfavorable because of the pseudoaneurysm’s giant size, compressive effect on the right ventricle, large thrombus burden, lack of distal PDA graft opacification, and need for revascularization of viable ischemic myocardium. In addition, involvement of the graft body extending toward the distal anastomosis limited the availability of suitable landing zones for covered stent placement.

**Figure 2:**
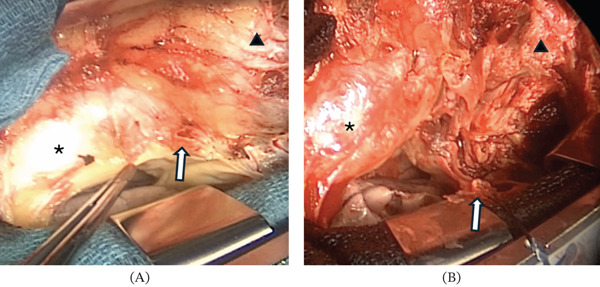
(A) Intraoperative image of the SVG pseudoaneurysm (triangle), ascending aorta (asterisk), and SVG graft as it arises from the ascending aorta. The SVG has been ligated, with the proximal portion held by the instrument and the distal nonaneurysmal segment marked by the white arrow. (B) Image of the wall of the SVG after it was opened.

**Figure 3:**
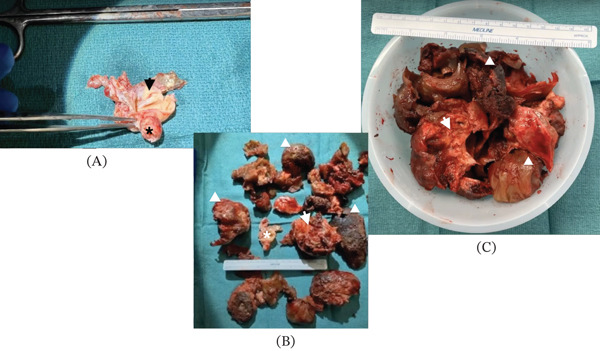
(A) Image of the true lumen of the proximal SVG (asterisk) and pseudoaneurysm capsule (black arrow). (B) Proximal SVG (asterisk), pseudoaneurysm capsule (white arrow), and representative thrombus of varying age (triangle). (C) Pseudoaneurysm capsule (white arrow) and representative organized thrombus of varying age (triangle).

Based on the preoperative imaging, we anticipated that sternal entry without breaching the pseudoaneurysm would be challenging because of its size and anterior location immediately beneath the sternum. Therefore, we planned to use hypothermic circulatory arrest in the event of uncontrollable bleeding. Cardiopulmonary bypass was achieved through cannulation of the right femoral artery and vein with 17 Fr and 25 Fr cannulas, respectively. During sternal entry, the pseudoaneurysm was breached, resulting in significant bleeding beneath the sternal plate. Circulatory arrest was initiated to minimize bleeding and facilitate vascular control of the graft inflow from the ascending aorta. Moderate hypothermic circulatory arrest at 25°C was deemed sufficient because we anticipated that sternal entry and cross‐clamping of the aorta would take approximately 10 minutes; this strategy also minimized rewarming time. Cerebral oximetry was monitored via near‐infrared spectroscopy throughout the case. Rapid and careful entry into the chest was accomplished. The proximal SVG graft was isolated, divided (Figure 2A), and clamped with a bulldog vascular clamp within 8 minutes of circulatory arrest. A disease‐free portion of the ascending aorta was identified for placement of the aortic cross‐clamp. Cardiopulmonary bypass was then resumed, antegrade cardioplegia was administered because the patient did not have aortic valve insufficiency, and the patient was subsequently rewarmed. The SVG pseudoaneurysm was opened (Figure 2B), and a large amount of thrombus of varying ages was evacuated (Figure 3). The pseudoaneurysm involved the SVG‐to‐PDA graft body and extended toward the distal anastomosis. The affected inferior wall myocardium appeared viable but demonstrated early ischemic changes such as tissue discoloration compared to well‐perfused surrounding myocardium. We proceeded with revascularization of the posterior descending artery and the diagonal artery using SVGs from the aorta because the diagonal artery was diseased on preoperative cardiac catheterization. After bypass, the myocardium was warm and healthy appearing, without evidence of wall motion abnormalities on intraoperative transesophageal echocardiography. The remainder of the operation was uneventful. Total cardiopulmonary bypass time and aortic cross‐clamp time were 213 and 69 minutes, respectively. Postoperative echocardiography showed improvement in the ejection fraction to 50%. The patient’s recovery was complicated by refractory seizures secondary to multiple embolic strokes and hypoxic‐ischemic encephalopathy, gastrointestinal bleeding, and hypoxic respiratory failure requiring tracheostomy. After a prolonged hospitalization, the patient’s tracheostomy was decannulated, and she was discharged 8 weeks postoperatively to a rehabilitation unit with intact motor function in all extremities. At 2‐week and 1‐month postoperative follow‐up, the patient’s cardiopulmonary, neurological, and gastrointestinal symptoms had resolved.

## 3. Patient Timeline

The patient underwent multivessel CABG 10 years before presentation. She presented with acute chest pain and was found to have a large SVG‐to‐PDA pseudoaneurysm with right ventricular compression and no distal graft runoff. After diagnostic CT, echocardiography, and coronary angiography, she underwent urgent redo sternotomy with femoral cardiopulmonary bypass, moderate hypothermic circulatory arrest, pseudoaneurysm resection, and revascularization of the PDA and diagonal arteries. Her postoperative course was complicated by seizures, embolic strokes and hypoxic‐ischemic encephalopathy, gastrointestinal bleeding, and respiratory failure requiring tracheostomy. She was decannulated and discharged to rehabilitation 8 weeks postoperatively with intact motor function and later experienced resolution of cardiopulmonary, neurologic, and gastrointestinal symptoms.

## 4. Discussion

SVG aneurysmal dilation may represent either a true aneurysm or a pseudoaneurysm. True aneurysms involve all layers of the vessel wall, whereas pseudoaneurysms represent a contained rupture and are reported to be approximately six times more common [1]. SVG aneurysmal dilation is a rare postoperative complication, with an incidence of less than 1%. These lesions rarely occur after CABG and can be classified according to the timing of presentation and the presence or absence of pseudoaneurysm. Early aneurysms occur less than 12 months after surgery and can have various etiologies, including graft infection and technical error related to conduit harvesting, preparation, or anastomosis. Late aneurysm formation occurs more than 5 years after the initial procedure and is thought to be related to atherosclerotic degeneration from persistent exposure to high arterial pressure [2]. SVG aneurysms usually involve the body of the graft, whereas pseudoaneurysms usually occur at the anastomotic site. The most commonly affected pseudoaneurysm site is the RCA graft (47%), followed by the left circumflex artery (29%) and the LAD artery (20%) [3]. SVG pseudoaneurysms occur approximately 12 years after bypass surgery and average 6.0 to 8.0 cm in diameter [4].

There is no consensus on the optimal treatment for patients with SVG pseudoaneurysms. The goal of treatment is to reduce the risk of complications and improve long‐term survival. Medical management for asymptomatic patients involves antiplatelet and antithrombotic therapy to prevent distal embolization. Most patients, however, ultimately require endovascular or open surgical repair because of the risk of rupture and associated mortality [5]. Once an SVG pseudoaneurysm is detected, size, patency, and anatomic location are among the factors that guide management. Repair can be performed with percutaneous catheter‐based interventions, including coil embolization or covered stent placement, or by median sternotomy with ligation of the pseudoaneurysm and revascularization [6]. Endovascular management may be feasible in selected patients with favorable graft anatomy, preserved distal runoff, and smaller pseudoaneurysms, including some reported SVG–RCA pseudoaneurysms measuring less than 6 cm [7]. Surgical repair can be challenging because of the need to safely enter the chest, particularly in patients with large pseudoaneurysms, carefully dissect around the pseudoaneurysm to avoid injury to adjacent structures, control bleeding, and prevent perioperative myocardial infarction from distal embolization of intraluminal thrombus [5, 8]. The location of the pseudoaneurysm along the SVG is also important, because manipulation of a proximal lesion may increase the risk of embolization of plaque contents into the ascending aorta and subsequent stroke. Kubota et al. reported a case of a large proximal SVG aneurysm managed with ligation and bypass during ventricular fibrillation. Instead of aortic cross‐clamping and cardioplegic arrest, they ligated the SVG inflow during a state of near‐zero flow on cardiopulmonary bypass [9]. The exact timing of repair is unknown, but prompt repair with resection and revascularization for symptomatic patients and for asymptomatic patients with large pseudoaneurysms has been shown to be an effective strategy with good long‐term outcomes [9].

This case underscores the challenges involved in the management of a large substernal SVG pseudoaneurysm, namely its size and location, the need for careful sternal entry, and the importance of a deliberate circulatory arrest strategy. This patient presented with a large symptomatic SVG pseudoaneurysm causing right ventricular compression without hemodynamic instability. Although there were no acute ECG changes or hemodynamic instability, the elevated troponin level, absence of distal PDA graft runoff, and intraoperative appearance of early inferior‐wall ischemic change suggested graft‐related myocardial ischemia rather than primary type 1 myocardial infarction. Given the pseudoaneurysm’s large size and location immediately beneath the sternum, breach of the pseudoaneurysm was likely unavoidable; therefore, careful sternal reentry, peripheral cannulation for cardiopulmonary bypass, and moderate hypothermic circulatory arrest to control hemorrhage before aortic cross‐clamping were crucial. Cooling to 25°C was adequate because the time needed to obtain vascular control of the SVG pseudoaneurysm was short, and this approach minimized subsequent rewarming time. However, if a longer period is anticipated for vascular control and isolation of the pseudoaneurysm, cooling to 14°C to 20°C can be considered to allow for a longer safe arrest time. In this case, sternal reentry and control of the bleeding source were achieved in approximately 10 minutes; however, adequate preoperative planning regarding peripheral cardiopulmonary bypass cannulation, hypothermic circulatory arrest, and operative repair remains essential. For patients with similar pseudoaneurysm morphology, early cardiopulmonary bypass via femoral cannulation and hypothermic circulatory arrest should be considered as part of the operative plan.

SVG aneurysms and pseudoaneurysms after CABG are rare but can have devastating complications if left untreated. Open ligation of the pseudoaneurysm with revascularization can provide good outcomes but may be technically challenging because of pseudoaneurysm size and location.

## Funding

No funding was received for this manuscript.

## Consent

Written informed consent for publication was not obtained. The case was reviewed by the institutional privacy/ethics office, which determined that the report contained de‐identified information and did not require written authorization/consent for publication. All direct identifiers and dates were removed, and nonessential details were omitted or generalized where possible, and only clinically necessary information is presented.

## Conflicts of Interest

The authors have no conflicts of interest.

## Data Availability

Data are available upon request from the authors.
